# In Vitro Blood–Brain Barrier-Integrated Neurological Disorder Models Using a Microfluidic Device

**DOI:** 10.3390/mi11010021

**Published:** 2019-12-24

**Authors:** Jin-Ha Choi, Mallesh Santhosh, Jeong-Woo Choi

**Affiliations:** 1 Department of Chemical and Biomolecular Engineering, Sogang University, 35 Baekbeom-ro (Sinsu-dong), Mapo-gu, 121-742 Seoul, Korea; jinhachoi@sogang.ac.kr; 2 Center for Integrated Biotechnology, Sogang University, 35 Baekbeom-ro (Sinsu-dong), Mapo-gu, 121-742 Seoul, Korea; santhosh@sogang.ac.kr

**Keywords:** neurological disorders, blood–brain barrier (BBB), microfluidic device, in vitro model, neuroinflammation

## Abstract

The blood–brain barrier (BBB) plays critical role in the human physiological system such as protection of the central nervous system (CNS) from external materials in the blood vessel, including toxicants and drugs for several neurological disorders, a critical type of human disease. Therefore, suitable in vitro BBB models with fluidic flow to mimic the shear stress and supply of nutrients have been developed. Neurological disorder has also been investigated for developing realistic models that allow advance fundamental and translational research and effective therapeutic strategy design. Here, we discuss introduction of the blood–brain barrier in neurological disorder models by leveraging a recently developed microfluidic system and human organ-on-a-chip system. Such models could provide an effective drug screening platform and facilitate personalized therapy of several neurological diseases.

## 1. Introduction

Neurological disorders (NDs), which are diseases of the central nervous system (CNS) and peripheral nervous system (PNS) such as Alzheimer’s disease (AD) and Parkinson’s disease (PD), have been on the increase whilst threatening the quality of life of affected individuals, especially the elderly, and their families [1,2,3,4,5]. There are many causes of NDs, but their origin and trigger have not yet been identified. Moreover, no therapeutic agents that can repair neural network damage caused during NDs in both CNS and PNS are available to date [6,7,8], although high costs and manpower have been invested in the development of new drugs. Therefore, development of effective drugs against each ND is urgently needed. In the drug development process, one of the significant factors is the development of effective drug-screening models, which would help lower costs and time. Generally, the main models for drug screening can be categorized into two systems: in vitro cell-based model and in vivo animal model. However, there are some critical limitations associated with using models as an alternative to an actual human system. The in vitro cell-based model has numerous gaps in comparison with an actual human biological system, such as bloodstream with an appropriated shear stress as well as a nutrient and oxygen delivery mechanism. Contrastingly, the in vivo animal model could not precisely emulate the human physiological system owing to species differentiation [9,10,11]. Moreover, there are severe ethical issues arising from the use of animals for screening drug candidates. Hence, a novel model platform is urgently warranted to enable the replacement of or overcome the shortcomings of both the aforementioned models.

The concept of the neurovascular unit (NVU) has emerged to further study neuronal circumstances and NDs [12,13,14,15]. An NVU comprises neuronal cells, predominantly encompassing neurons, astrocytes, pericyte, and the surrounding blood endothelial cells. These cells and conditions, such as fluidic flow and inflammatory state, should be considered as a unit or organ for analyzing the etiological approach and drug screening more accurately. In fact, brain endothelial cells and neuronal cells could affect each other by crosstalk via chemokine and cytokine secretion [16,17,18]. Moreover, fluidic flow could induce the differentiation of blood endothelial cells owing to its shear stress [19,20,21]. Neurological studies have revealed that there are several differences between a single neuronal cell and NVU [22,23,24,25]. From this point of view, ND studies have been conducted to identify an in vitro NVU that would mimic the actual NVU of a human body. For emulating an NVU in vitro, a microfluidic device should be integrated for simulating fluidic flow mimicking the bloodstream in the vasculature. In this review, in vitro neuronal models and blood–brain barrier (BBB) models are introduced. Subsequently, microfluidic-integrated NVU models will be briefly introduced with the trials of reconstruction of NDs, such as AD and general neuroinflammation in vitro. In this regard, each section in the review individually focuses on one of the following in vitro models: BBB model, ND model, and ND model with microfluidic device. 

## 2. In Vitro Blood–Brain Barrier (BBB) Model with a Microfluidic Platform 

### 2.1. The Concept of the BBB

The BBB is a dynamic boundary between the brain and the bloodstream, which separates blood fluid from the CNS, including the brain and spinal cord [26,27]. The BBB provides a physical and biological barrier that controls passive and active transport [28]. The physical barrier is established by vascular endothelial cells that are connected by tight junction proteins, including zonula occludens 1 (ZO-1) and claudin [29]. These proteins induce limited diffusion of ions and several biomolecules through paracellular pathways. In detail, the BBB is a biological barrier for the CNS, and it selectively transports diverse biomolecules such as essential nutrients and potentially harmful metabolic products. In the case of selective transportation, the biomolecules that want to enter in CNS are required to take the transcellular route through individual vascular endothelial cells. Only small lipophilic molecules such as exosomes can pass through the BBB without any restrictions, and several prospective drug candidates fail to overcome the BBB and its efflux transporters. There are two main transporters of the BBB, ATP-binding cassette (ABC), which includes ABCG2/BCRP, ABCB1/MDR1, ABCA2, ABCA8, ABCC1, ABCC4, and ABCC5, and solute-like carrier (SLC), which includes SLC7A5/LAT1, SLC2A1/GLUT1, and SLC16A1/MCT1 in a real BBB system [30]. Thus, vascular endothelial cells and their tight junction are the core components of the BBB in the CNS. In addition, pericytes and astrocytes, basement membranes, and extracellular matrix (ECM) also play crucial roles in preserving the function of the BBB [31,32,33]. If the BBB permeability increases and dysregulation of influx and efflux occurs, toxic materials and immune cells from the blood vessels penetrate the CNS. This damage has been correlated with various NDs, including AD, PD, and multiple sclerosis [34]. Therefore, the development of relevant in vitro platforms that can precisely emulate BBB phenotypes and enable monitoring of a change in BBB integrity and neuronal degeneration could advance our understanding of NDs and facilitate new drug development. An ideal in vitro BBB model should possess some essential features of the human BBB. First, three-dimensional vessel-like structures should be made using vascular endothelial cells originating from brain tissues. Additionally, co-culturing should be possible for inducing cell–cell interactions between vascular endothelial cell and pericytes or astrocytes. Third, fluidic flow ought to be presented on the vascular endothelium with similar shear stress. To date, co-culture and shear stress have been successfully exhibited in several in vitro BBB models with microfluidics. Those microfluidic models certainly overcome some limitations of traditional two-dimensional culture models including different cell morphologies, differentiation levels, and gene expressions [35]. However, there are still some challenging aspects pertaining to mimicking of BBB in vitro, namely varied cell lines, cell differentiation, homeostasis, immune reaction, tissue maintenance, and cell structural support. In this section, several in vitro BBB models categorized into static and microfluidic-integrated systems will be briefly discussed (Table 1). 

### 2.2. Static BBB Model

**Figure 1:**
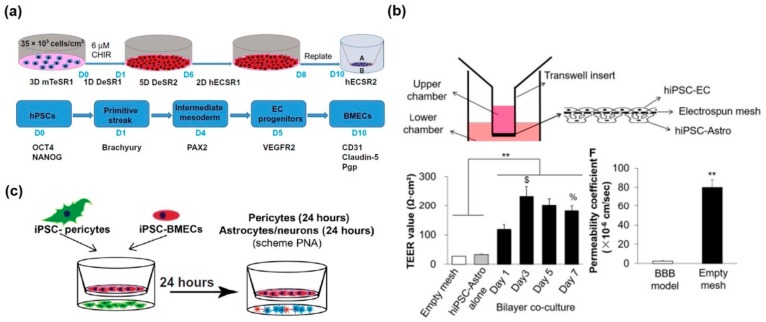
In vitro static blood–brain barrier (BBB) models. (**a**) 2D Transwell-based BBB model by human pluripotent stem cell (hPSC)-derived cells. Reproduced with permission from [37]. Copyright 2017, American Association for the Advancement of Science (AAAS). (**b**) 2D nanofiber-based BBB model by 3D printed holder and electrospun poly(lactic-*co*-glycolic acid) (PLGA) mesh and human induced pluripotent stem cell-derived cells for BBB. Reproduced with permission from [38]. Copyright 2018, ACS Publications. (**c**) 2D Transwell-based BBB model with isogenic human model by iPSCs. Reproduced with permission from [40]. Copyright 2017, American Association for the Advancement of Science (AAAS).

For the development of the in vitro BBB model, many researchers have utilized the membrane-integrated static cell culture system, which consisted of a larger well and inserted smaller well with the porous membrane, for separating blood vessels from the neural site. The advantages of the Transwell-based static model are ease of use and high-throughput drug delivery and penetration assay. In general, the Transwell system has been frequently applied to form the BBB structure with appropriate vascular endothelial cells such as human umbilical vein endothelial cells (HUVEC), human cerebral microvascular endothelial cells (hCMEC), and primary human-derived vascular endothelial cells [36,37,38]. Immortalized cell lines exhibit a loss of endothelium-specific properties, including loss of tight junctions yielding subphysiologic transendothelial electrical resistance (TEER). In addition, it is difficult to obtain human primary vascular endothelial cells in sufficient quantities for drug screening and disease model development, and thus, the procedure cannot be readily expanded to a larger scale. Recently, some attempts have been made to differentiate human pluripotent stem cells (hPSCs) to vascular endothelial cells in an in vitro BBB model for achieving the relevant physiology by recapitulation of the key signaling pathways in vivo. Qian et al. constructed an in vitro BBB model using hPSC-derived vascular endothelial cells and the Transwell system [39] (Figure 1a). The authors claimed that differentiated vascular endothelial cells expressed specific markers, formed BBB, and exhibited efflux transporter properties. Moreover, these cells showed tube formation, low-density lipoprotein uptake, and high TEER value of >3000 ohm·cm^2^, similar to the vascular endothelial cells in vivo. Qi et al. also used human induced pluripotent stem cells (iPSCs) that differentiated into two different kinds of cells, astrocytes and vascular endothelial cells [40] (Figure 1b). For mimicking the human microphysiological BBB system more closely, poly(lactic-co-glycolic) acid (PLGA) nanofiber was used to form a nanofibrous mesh on a 3D printed holder. Using this porous structure, differentiated astrocytes and endothelium were co-cultured under static conditions in the presence of a strong BBB. When applying anti-glioblastoma multiforme (GBM) drugs and the neurotoxic peptide [amyloid-beta (Aβ) 1-42], TEER values were decreased, but the barrier functions were maintained to a certain extent. For quantifying transendothelial delivery of nanoparticles, De Jong et al. developed a filter-free in vitro BBB model using a collagen gel on a conventional well plate, which was covered with an hCMEC/D3 cell monolayer [41]. The authors indicated that there are limitations to the testing of transendothelial delivery of nanoparticles using an extracellular matrix-coated porous membrane filter owing to the adhesion of numerous types of nanoparticles in a membrane filter and within the filter pores. This filter-free BBB model allowed for high-throughput quantitative measurement of transendothelial transport of nanocarriers via fluorescence spectroscopy. Through this platform, nano-sized polymersomes showed 6.6% ± 2.2% transcytosis. Using this platform, the transendothelial transport of drug-loaded nanoparticles could be evaluated in a setting that more closely mimics the human BBB in vivo. Stebbins et al. demonstrated that pericytes play important roles in the formation and physiological function of BBB [42] (Figure 1c). Therefore, the brain pericyte-like cells, differentiated from the neural crest stem cells (NCSCs), were co-cultured with endothelial cells and neuronal cells (astrocytes and neurons). They asserted that the brain pericyte-like cells induced BBB properties in brain microvascular endothelial cells (BMECs), including barrier enhancement and transcytosis reduction. Furthermore, brain pericyte-like cells were incorporated with iPSC-derived BMECs, astrocytes, and neurons to form an isogenic human NVU model, which is useful for ND studies and drug development. However, these static models could not precisely mimic the human BBB system because some key elements, such as blood flow, were missing. 

### 2.3. In Vitro Microfluidic-Integrated BBB Model

**Figure 2:**
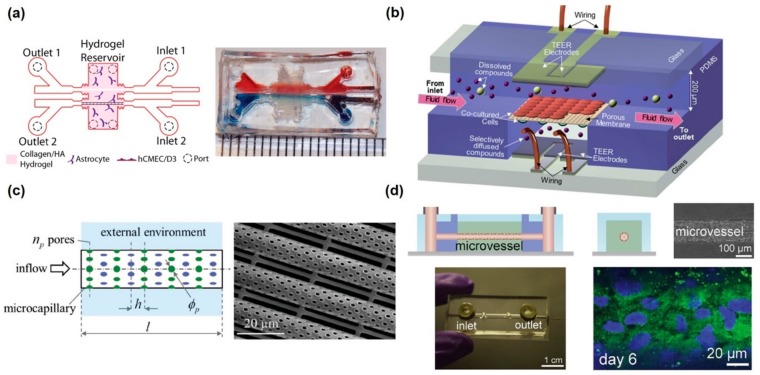
In vitro microfluidic-integrated BBB models. (**a**) Horizontal-aligned microfluidic BBB model with 3D hydrogel structure for induction of crosstalk between neuronal cells and endothelium. Reproduced with permission from [43]. Copyright 2017, Elsevier. (**b**) Vertical-aligned microfluidic BBB model with porous membrane for the separation of two channels and transendothelial electrical resistance (TEER) electrodes. Reproduced with permission from [44]. Copyright 2012, The Royal Society of Chemistry (RSC). (**c**) 3D tubular structure-based BBB model with porous tube (mimicking a microcapillary) that simultaneously scaffolds the cells and allows for species transport toward the external environment. Reproduced with permission from [49]. Copyright 2017, WILEY-VCH. (**d**) Human induced pluripotent stem cells (iPSCs)-derived blood–brain barrier microvessels by the wire removal method. Reproduced with permission from [50]. Copyright 2019, Elsevier.

There have been in vitro BBB models with microfluidic devices in order to add similar functions of the in vivo BBB system. The actual BBB is positioned between blood flow and the neural tissue and prevents the transport of biomolecules. Thus, microfluidic flow with vascular endothelial cells should be essential to mimic the human BBB and its physiological functions. For the observation of barrier function and transcytosis of molecules, the BBBs were positioned in the middle of the channels and the divided channels were aligned horizontally or vertically. Prabhakarpandian et al. developed a simple microfluidics vasculature model of the BBB with a horizontal-aligned structure [43]. Two spaces, the apical and basolateral side were positioned horizontally and divided by the micropillar with 3 µm gap. At the apical side, vascular endothelial cells were maintained, and they blocked the FITC–dextran permeation from the apical side to the basolateral side. In this system, upregulation of tight junction proteins was observed by Western blot analysis of ZO-1 and claudin-1. Deosarkar et al. also exhibited a similar strategy, consisting of a tissue chamber at the center and a surrounding vascular channel for applying fluidic flow [44]. The authors claimed that the developed BBB chip not only allows for culturing of brain endothelial cells under shear flow that mimic the microvessels in vivo, but also permits interactions between the endothelial cells and astrocyte in the middle chamber. Partyka et al. showed a 3D model of the BBB, composed of two horizontal channels and a hydrogel reservoir at the center of the two channels [45] (Figure 2a). In this system, there were two specific functions that applied cyclic strain stimulation by the pressure and electrical system to measure the TEER value in situ. A pulsatile flow to the vessel channels induced pulsatile strain to the vascular wall, offering an opportunity to investigate stretch-induced transport. Through this platform, pulsatile wave facilitates retrograde transport of high molecular weight dextran along the basement membrane between the basal endothelium and astrocytes.

Vertically aligned microfluidic channels have also been utilized to develop an in vitro BBB system. Booth et al. developed a microfluidic BBB model comprising two perpendicular flow channels and two TEER electrodes with a comparatively thin culture membrane (10 µm) [46] (Figure 2b). This microfluidic BBB model showed significantly higher TEER levels than the static models, with a comparatively short time to steady-state TEER; real-time TEER measurement provided information regarding transient effects of histamine exposure. For testing the effectiveness of several drugs against NDs, a similar BBB model was used to measure the TEER value and quantify drug permeation from the luminal to the abluminal side via liquid chromatography (LC) [47]. Dynamic models, which applied fluidic flow with 15 dyne/cm^2^ shear stress to mimic in vivo BBB, produced a significantly higher average TEER and lower drug permeability than static models. Additionally, astrocyte and vascular endothelial cell co-culture models exhibited a higher average TEER and lower drug permeability than monoculture models. Shao et al. also demonstrated drug permeability by performing drug screening and drug-induced cytotoxicity by constructing a microfluidic BBB model, which comprised hCMECs for BBB formation and glioma cells (U251) in a 3D hydrogel [48]. Through this platform, BBB permeability and antitumor activity of CNS drug candidates could be simultaneously evaluated. The authors claimed that this device enables rapid analysis of drug candidates and accelerates drug development. Bonakdar et al. developed a microfluidic platform with electrical equipment for real-time measurement of BBB permeability via estimation of the pulsed electric field effect [49]. Consequently, pulsed electric fields enhanced drug delivery by disrupting the integrity of BBB and allowing impermeable drugs to translocate through BBB. Increased permeability of the BBB model at sub-electroporation pulses suggests that permeabilization is induced by the paracellular pathway via opening of tight junctions.

Actual blood vessels in the neurological system are in the form of 3D tubes, which enables them to establish greater contact with neural tissues and interact with them as opposed to a 2D model. Vascular endothelial cells tend to engulf the inner channels or form tubular structures and BBB structures. Thus, several attempts have been made to construct an in vitro BBB using artificial channels. Kim et al. developed a 3D in vitro brain microvasculature system embedded within the bulk of a collagen matrix [50]. To construct a BBB, a microneedle was applied to the 3D collagen matrix to form a channel. The cylindrical collagen microchannels, created using a microneedle, were surrounded by vascular endothelial cells, and the developed BBB was utilized for testing the permeability and cytotoxicity of hyperosmotic mannitol, which causes disruption of the barrier function. Moreover, recovery of the barrier function was also exhibited after four days. Marino et al. constructed a microtubular structure inspired by the brain capillaries via two-photon lithography, and it was used as a basement membrane of the artificial BBB, which had been formed by coculturing vascular endothelial cells and glioblastoma cells [51] (Figure 2c). This platform was constructed in line with the actual dimensions of a human BBB and showed the maturation of tight junctions and excellent BBB performance, including prevention of transcytosis of biomaterials and high TEER value. The main advantage of two-photon lithography is that the pore size, pore density, and capillary diameter can be easily controlled. Linville et al. utilized iPSC-derived vascular endothelial cells to construct BBB with 3D ECM gel and a microstructured channel via microwire elimination after ECM hardening [52] (Figure 2d). Vascular endothelial cells reached quiescence and led to the development of a uniform basement membrane and tight junction protein within six days. Furthermore, several BBB functions such as inhibition of permeability or leukocyte adhesion were tested using a P-glycoprotein (P-gp) inhibitor or via cytokine activation, respectively. Besides construction of a tube-shaped microchannel, spheroids were formed for studying the in vitro BBB model [53,54]. The spheroid core consists of neural cells including astrocytes, whereas vascular endothelial cells and pericytes cover the neural cell surface and act as BBB, which in turn regulates biomolecule transport. Compared with the 2D-Transwell system, the spheroid model of BBB showed higher expression of tight junction proteins, VEGF-dependent permeability, efflux pump activity, and receptor-mediated transcytosis of angiopep-2. The authors claimed that this BBB spheroid model is easily scalable to high-throughput capacity owing to its ease of use and low costs involved in the development of spheroids. 

## 3. In Vitro Neurological Disorder Models 

The CNS is one of the most complex, highly compartmentalized, and layered organ systems consisting of diverse cell types; its functional connectivity is established through axons and dendrites [55]. Various animal models were developed to study and understand brain function and related diseases [56]. Even though these models provide valuable insights into the CNS and its related disease, they are limited by high costs, labor-intensive procedures, and low-throughput [57]. These limitations have driven scientists to develop high-throughput, low-cost, and simple in vitro ND models. Technological advancement over the past decade in the field of microfluidics [58] and micro-electromechanical systems have further fueled the development of in vitro CNS and related disease models. In this section, we summarize some of the currently developed microfluidic platforms for CNS disease models (Table 2).

### 3.1. 2D Microfluidic ND Models

Compared with the conventional cell culture methods, microfluidic cell culture methods possess several attractive features, including continuous nutrient supply, less liquid consumption, ease of handling, less time consumption, and low costs. Hence, microfluidic systems are invaluable and versatile tools for cell-based assays [59,60]. Compartmentalized microfluidic systems (CMSs) [61,62] composed of multiple chambers separated by microgroove arrays, which allows the specific isolation of subcellular components (e.g., segregated axons and soma of the neurons) are particularly useful for studying neuron–neuron interaction, synapse formation, axon signaling, myelination, and other important parameters that are essential for understanding NDs [63,64]. 

The spinal cord is an essential and complex part of CNS, which serves as the path for motor and sensory neurons. Spinal cord injuries (SCIs) arise when its complex structure is damaged by external forces, thereby resulting in total or partial loss of its function [65]. 2D CMSs are one of the useful platforms to study such kinds of SCIs. Several 2D in vitro microfluidic device models have been developed for studying and examining axonal injury. All of the microfluidic platforms utilize multiple compartments and adopt various injury mechanisms or approaches, including vacuum-assisted [66,67], physical (stretch and strain), and chemical-based injuries [68] for studying numerous physical and biochemical factors involved in SCIs and for developing therapeutic agents to treat such conditions.

**Figure 3:**
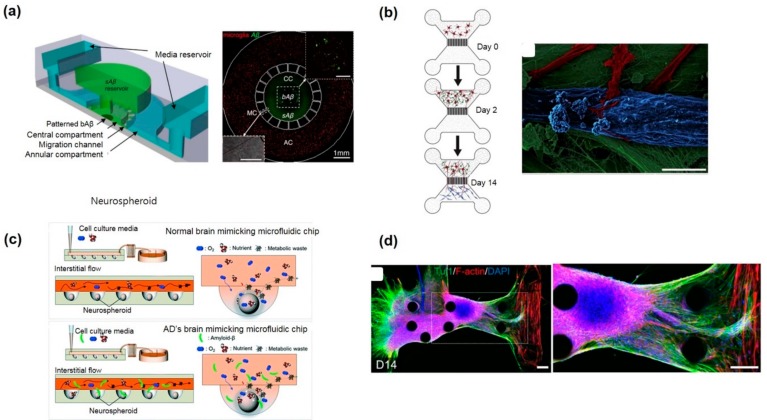
In vitro neurological disorder models. (**a**) 2D microfluidic Alzheimer’s disease (AD) platform to study microglial responses to various types of Aβ. Reproduced with permission from [67], copyright 2013 under creative common license (attribution—noncommercial). (**b**) 2D compartmentalized microfluidic neuromuscular junction (NMJ) model to study amyotrophic lateral sclerosis (ALS), reproduced with permission from [70], copyright 2013, Elsevier. (**c**) A hydrogel free 3D neurospheroid-based AD model congaing concave microwell to generate a neurospheroid and study neurotoxicity of Aβ. Reproduced with permission from [75] copyright 2017, The Royal Society of Chemistry (RSC). (**d**) Neural outgrowth and formation of a human motor unit along with NMJ in a 3D ALS motor unit model and NMJs in microfluidic devices reproduced with permission from [79]. Copyright 2018, AAAS.

Besides the axonal injuries, 2D CMSs have also been used for studying the pathophysiology of various neurodegenerative diseases and for investigating their feasibility as a drug screening tool. For instance, Cho et al. used a microfluidic chemotaxis platform for studying AD. This platform facilitates the study of microglial accumulation in response to week-long gradients of soluble Aβ and patterns of surface-bound Aβ. This study helps improve the elucidation of microglial migration and provides insights into the pathophysiology of AD [69] (Figure 3a). In another CMS-based AD model, Song et al. demonstrated and provided evidence for the spreading of Aβ through neuronal connections [70]. In another example, Kunze et al. studied the pathogenesis of amyotrophic lateral sclerosis (ALS), which is a common motor neuronal disease, by co-culturing neurons and astrocytes with wild-type or mutated superoxide dismutase enzyme 1 (SOD1) for demonstrating oxidative stress on cortical neurons [71]. For the first time, Southam et al. constructed a co-culture system for forming an excellent neuromuscular junction (NMJ) for studying ALS. They revealed that the elongated axons come in contact with myotubes and also demonstrated the role of glial cells in motor neuron spreading [72] (Figure 3b). Such kinds of 2D microfluidic ND models are advantageous owing to their ease of application and ease of quantification as well as owing to their integrated optical and electric stimulation; thus, their use can be further explored in future studies for discovering therapeutic agents against NDs.

### 3.2. 3D Microfluidic Neurological Disorder Model

The neuroscience research field is revolutionizing owing to the advances in 3D neuronal culture models derived from human stem cells [73]. These models help in a reliable recapitulation of in vivo cytoarchitecture as opposed to the conventional 2D and animal models. There are two typical approaches that can be adopted for creating 3D neural models: brain organoids and brain-on-a-chip technology [74,75]. Recently, these technologies have been combined to generate a new 3D model of organoids-on-a-chip [76]. These technologies have been utilized recently by numerous scientists globally to generate new reliable ND models.

Park et al. developed a 3D AD model on a chip which consists of a concave array of microwell for forming homogeneous neurospheroids. They tested the toxic effect of Aβ on neurospheroids, and for the first time, they proposed a 3D microfluidic in vitro brain model for neurodegenerative disease and high-throughput drug screening [77] (Figure 3c). Recently, a 3D human AD triculture system containing neurons, astrocytes, and microglia in a 3D microfluidic platform was developed by Park et al. [78] This model possesses most of the key AD features such as Aβ aggregation, p-tau accumulation, and neuroinflammatory activity such as microglia recruitment and neurotoxic activity (including axonal cleavage). A majority of AD features have been recapitulated by this model in a single microfluidic platform to date. 

PD is a complex neurodegenerative disorder characterized by severe loss of nigrostriatal dopaminergic fibers. In a recent study, a 3D microfluidic platform was utilized to cultivate PD patient-specific dopaminergic neurons. In contrast to 2D culture, 3D approaches reveal robust endophenotypes affecting only dopaminergic neurons. This model has the potential to develop patient-specific therapeutic drugs against the disease [79]. In a recent study, 3D co-culture and compartmentalization of mouse embryonic stem cells (mESCs)-derived motor neurons and skeletal muscle cells within an ECM were utilized to create NMJs [80]. The optogenetic approach was used to facilitate MN excitation. This model can be extended to study NDs including ALS. The optogenetic approach was used to facilitate MN excitation. This model is extended in the latter study to mimic the neurological disorder ALS by the same group [81] (Figure 3d). Human iPS-derived muscle cells along with the optogenetic motor neuron derived from ALS patient were utilized to realize ALS disease on-chip. ALS motor neurons generated fewer muscle contractions and motor neuron degradation compared to non-ALS chips.

## 4. In Vitro BBB-integrated Neuronal Models

### 4.1. In Vitro Neurovascular Unit Models 

As previously discussed, the BBB comprises an interface between peripheral circulation and the CNS. The main functions of the BBB are not only nutrient supply to the CNS and waste product removal but also the prevention of pathogens and release of toxic products from blood vessels [82]. However, most in vitro models of BBBs include only brain vascular endothelial cells and astrocytes in a static or fluidic culture system. Therefore, it has a significant limitation regarding the emulation of an actual in vivo human physiological system of neural systems and NDs. NVU consists of several cell types including vascular cells (endothelial cells, pericytes, and vascular smooth muscle cells), glia (astrocytes, microglia, and oligodendrocytes), and neurons [12]. In this concept, the BBB is part of the NVU that prevents uncontrolled materials and pathogens into the brain and mediates the exchange of molecules via a specialized substrate-specific transport system. The in vitro NVU system has been provided as a model to study interactions among the CNS neurons, the cerebrospinal fluid compartment, and circulating or resident immune cells including microglia. In this section, representative in vitro NVU models with their specific functions for emulating in vivo human NVU will be discussed; furthermore, details on the transcytosis of biomaterials or nanoparticles will be provided below (Table 3).

**Figure 4:**
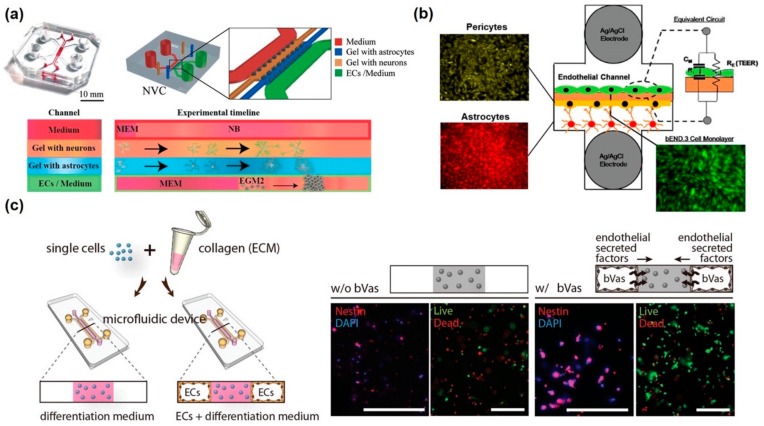
In vitro BBB-integrated neurovascular unit models. (**a**) Horizontal-aligned microfluidic neurovascular unit model with neuron, astrocyte, and endothelium. Reproduced with permission from [81]. Copyright 2017, The Royal Society of Chemistry (RSC). (**b**) Vertical-aligned neurovascular unit consisting neuron, astrocyte, pericyte, and endothelium with Ag/AgCl TEER electrodes. Reproduced with permission from [84]. Copyright 2016, ACS publication. (**c**) Horizontal-aligned neurovascular unit model with neural stem cells (NSCs) and endothelium for induction of crosstalk between NSC and endothelium. Reproduced with permission from [87]. Copyright 2014, WILEY-VCH.

Adriani et al. exhibited an in vitro 3D neurovascular microfluidic model comprising the BBB and neurons for mimicking the neurovascular model [83] (Figure 4a). In this system, the channels of media, neurons, astrocytes, and endothelial cells were aligned horizontally, and two neuronal channels were made up of collagen, similar to the actual 3D neuronal structure. Neurons could be quantitatively analyzed in terms of neuronal responses with neurite outgrowth and neuronal activity, whereas endothelial cells form BBB and perform specific functions, such as size-selective permeability, similar to the existing in vitro BBB models. Nguyen et al. developed a hybrid elastomer–plastic microdevice using amine-functionalized poly(dimethylsiloxane) (PDMS) to mimic the human BBB in vitro [84]. In brief, poly-dopamine was functionalized on the amine-functionalized PDMS to provide a stable surface for culturing vascular endothelial cells and astrocytes. Using this platform, the authors showed four characters for verifying the formation of BBB, NVU, tight junctions, actin filaments as well as the expression of tight junction protein (ZO-1) and the low permeability of FITC–dextran molecules. As opposed to the monoculture model of endothelial cells, coculturing of astrocytes and vascular endothelial cells revealed greater integrity of BBB, as estimated TEER value measurements and permeability test results, as well as higher F-actin expression. Brown et al. successfully fabricated the in vitro NVU model consisting of vascular space and a 3D brain parenchyma site [85]. A filter membrane was positioned between these two channels, and crosstalk between pericyte–astrocyte and endothelial cells was enabled. The perfusion channel was presented on the brain parenchyma channel to supply nutrients and apply some biomaterials. This dual perfusion nature of this device allows for the manipulation of either side of the BBB as well as differential delivery of drugs and nutrients to either the vasculature or brain chamber. A similar structure and composition of the in vitro NVU was revealed, consisting of pericytes, astrocytes, and vascular endothelial cells [86] (Figure 4b). In situ measurement of the TEER value was successfully taken by inserting two independent electrodes into two separate microfluidic channels. As expected, the tri-culture model, which consisted of pericytes, astrocytes, and endothelial cells, showed the highest TEER value and lowest permeability. Additionally, P-gp efflux pump was functionally expressed on the endothelial cells of bi- and tri-culture models. However, development of in vitro NVU was limited using stable cell lines because immortalized cells do not exactly or equivalently mimic the functions of actual endothelial cells and neuronal cells. 

Some attempts have been made to use neuronal cells and endothelial cells that had been differentiated from stem cells for developing NVU. Appelt-Menzel et al. established in vitro NVUs using iPSCs and induced multipotent stem cells (iMSCs) [87]. The authors mentioned that the developed neurovascular model showed a TEER value of 2500 Ω·cm^2^ and upregulation of BBB-related genes. Additionally, tight junction proteins were found to be present, and a reduction in TEER value was confirmed by treatment with claudin-specific tight junction modulators. Thus, the abovementioned neurovascular model can be personalized to specific individuals using their respective cells. Canfield et al. also constructed an NVU model comprising isogenic endothelial cells, astrocytes, pericytes, and neurons differentiated from iPSCs [88]. Barrier tightening induced by iPSC-derived astrocytes and neurons indicates their BBB-inductive capacity. However, such aforementioned systems involve the application of stem cell-derived cells to a traditional Transwell system, which could not mimic fluidic flow. Shin et al. developed a 3D neurovascular model, which consisted of vascular endothelial cells and neural stem cells on a 3D collagen structure with a microfluidic device [89] (Figure 4c). This microfluidic platform comprising 3D ECM and endothelial cells can elicit spatiotemporal control of the neural stem cells by generating a gradient of chemical factors and maximizing paracrine- and autocrine-signaling effects. Consequently, vascular endothelial cells could regulate self-renewal and differentiation of neural stem cells. In detail, they suppressed neuronal generation but promoted differentiation into astrocytes and oligodendrocytes. Using this platform, it was possible to observe how stem cells inside an NVU released secretomes from endothelial cells. Campisi et al. emulated the microvascular structure in 3D self-organized endothelial cells and surrounding pericytes [90]. The iPSC-derived vascular endothelial cells underwent angiogenesis to the 3D hydrogel, where iPSC-derived pericytes and astrocyte were maintained. These neuronal cells formed an actual neurovascular network, comprising small lumens (approximately measuring 70 µm in diameter) and showing low permeability and transport selectivity. Moreover, gene expression of membrane transporters, tight junction, and extracellular matrix proteins, was similar to the in vivo neurovascular models with equivalent geometrical structures. Using these in vitro NVU models with BBB, it is possible to test certain biological events such as transcytosis, angiogenesis, and neuronal disorder development. 

### 4.2. In Vitro Neurological Disorder Models

**Figure 5:**
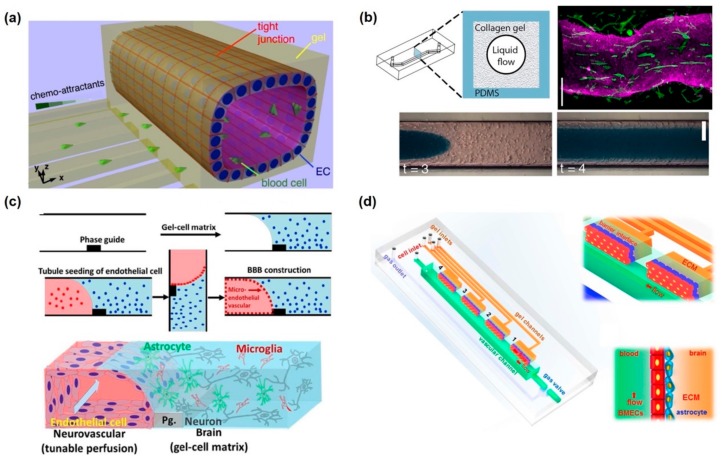
In vitro microfluidic-integrated neurological disorder models. (**a**) 3D model of BBB on a microfluidic platform with microglia cells for observation immune-reaction of the TNF-α-mediated neuroinflammation. Reproduced with permission from [94]. Copyright 2015, Springer Nature. (**b**) Tubular structured neurovascular unit by pressure-driven viscous fingering method. It is stimulated by TNF-α for emulating neuroinflammation. Reproduced with permission from [95]. Copyright 2016, PLOS. (**c**) Horizontal-aligned neurovascular unit for organophosphate (OP) toxicity screening by 3D tetra-culture for brain on chip. Reproduced with permission from [96]. Copyright 2018, Springer Nature. (**d**) Horizontal-aligned neurovascular unit for brain metastasis. Each unit of this device consists of four uniform BBB regions, one vascular channel, one gas channel, one gas valve and four gel channels. Reproduced with permission from [97]. Copyright 2016 Springer Nature.

Neuronal disorders, including AD, multiple sclerosis, PD, and ALS that negatively affect mental and physical functioning, occur in the CNS owing to the loss of neuronal structure and function [91]. Recent studies have recognized that the inflammatory process is closely related to multiple neurodegenerative pathways. Neuroinflammation is defined as the reactive response of the CNS against elements that interfere with homeostasis inside or outside the CNS and is characterized by increased glial activation, pro-inflammatory cytokine concentration, BBB permeability, and leukocyte invasion [92,93,94]. It may be initiated in response to a variety of cues, including infection, traumatic brain injury, toxic metabolites, or autoimmunity. As a result, the neuroinflammation process should affect neuronal cells in CNS and induce loss of cellular functions and in turn give rise to the symptoms of neuronal disorders. Therefore, it is important to mimic the neuroinflammatory state in a microfluidic neurovascular model for studying neuronal disorders. Achyuta et al. developed an in vitro NVU model comprising vascular endothelium, astrocytes, neurons, and microglia [95]. For inducing an inflamed state in this model, a pro-inflammatory cytokine, TNF-α, was added to the microfluidic chip, after which the barrier function of the BBB was found to decrease. Additionally, microglia and astrocytes were activated by TNF-α from the vascular channel. This phenomenon was shown to be the initial step of neuroinflammation. The authors claimed that this system could facilitate the delivery of nutrients, drugs, cells (immune or stem cells), nanomaterials, and microbes via vascular channels and enable the elucidation of its biological changes. Cho et al. established a 3D NVU model and induced neuroinflammation via TNF-α secretion and ischemia [96] (Figure 5a). Before achieving an inflamed state, BBB permeability was tested for transcytosis of FITC–dextran and neutrophils, and inhibition of penetration was observed. The purpose of inducing a neuroinflammatory state was to show the biological changes after treatment with drugs such as edaravone and Y27632. On administration of edaravone, an antioxidant, the expression of tight junction slightly increased from 46% to 53%. It was successfully demonstrated that this kind of NVU model could be utilized as a drug-screening platform against neuroinflammation. Herland et al. also constructed a 3D neuroinflammation model consisting of pericytes, astrocytes, and vascular endothelial cells. The cylindrical vascular channel was generated in collagen gel via a pressure-driven viscous fingering method [97] (Figure 5b). On application of this method, a normal 3D cell–cell interaction was observed, and neuroinflammation was also observed after stimulation with TNF-α. Measurement of the five pro-inflammatory cytokines, namely granulocyte colony-stimulating factor (G-CSF), granulocyte macrophage-colony stimulating factor (GM-CSF), interleukin-6 (IL-6), IL-8, and IL-17, revealed that neuroinflammation could be more closely mimicked in a living brain than in a static Transwell culture even when the same cells were co-cultured. 

Besides simulation of neuroinflammation, some studies emulated specific neuronal disorders, including AD and brain tumors. Koo et al. demonstrated the neurotoxic effect of organophosphate-based compounds, which could cause excitotoxicity, seizures, and brain damage via acetylcholinesterase inhibition [98] (Figure 5c). For toxicity screening, the 3D NVU model consisted of two horizontally aligned channels: a vascular fluidic channel and a 3D gel–cell-matrix without an artificial membrane. The results revealed that organophosphates infiltrated BBB and rapidly inhibited acetylcholinesterase activity and that in vitro toxicity correlated with in vivo toxicity. Xu et al. developed an organotypic NVU model to investigate metastatic brain tumors [99] (Figure 5d). Some malignant cancer cell lines, including MDA-MB-231 and A549, were found to show strong transcytosis from blood vessels to brain tissues on application of the aforementioned model. Additionally, the model enables visualization of morphological and phenotypical changes of vascular endothelial cells as well as quantitative evaluation of various drugs against tumor cells. As previously described, Park et al. constructed an in vitro model of AD using a 3D brain-on-a-chip technology; this model consisted of neurospheroids and interstitial flow, which mimicked the environment of an actual brain [77]. Neurospheroids cultured while maintaining interstitial flow were larger and formed a more robust and complex neural network than those cultured under static conditions. For AD induction, Aβ peptides were continuously treated with neurospheroids while maintaining an appropriate flow rate; this model showed reduced viability of neurospheroids and caused significantly more destruction of neural networks, whereas the static model showed better viability. As a cost-effective alternative method to animal testing, such microfluidic-based NVU models can be scaled to high-throughput for screening potential drugs to treat neuronal disorders, even though they need to be developed using some crucial elements, including immune cells, and under in vivo-like physiological circumstances. 

## 5. Future Perspective and Conclusions

For treating neuronal disorders, it has been reported that effective novel therapeutic agents have been developed and applied to pre-clinical and clinical testing [100,101,102]. However, the development of new drugs is sluggish because of it being a time-consuming and costly process and owing to the use of inappropriate models, including in vitro static models and in vivo animal models. Therefore, microfluidic-integrated in vitro NVU models, including BBB functionality, are utilized for assessing drug efficacy by factoring in parameters such as delivery efficiency and successful treatment rate. In this regard, the microfluidic-integrated NVU models are seen to perform two essential functions. First, BBB transcytosis caused by drug candidates needs to be evaluated. Several attempts have already been made to measure the transcytosis or penetration of diverse therapeutic agents through the BBB. In particular, surface-functionalized nanoparticles accompanied by encapsulation or surface-modification of drugs are an emerging technology for penetrating the BBB [103,104,105]. Chai et al. developed red blood cell membrane-coated nanoparticles with a CDX peptide, which is derived from candoxin and shows a high-binding affinity with nicotinic acetylcholine receptors on the surface of the endothelial cell [106]. Doxorubicin-loaded cell membrane-coated nanoparticles have superior therapeutic efficacy and markedly reduce toxicity to neural cells as opposed to a nontargeted drug. Fan et al. showed that heavy-chain ferritin (H-ferritin)-modified iron oxide nanocarriers were transported across the intact BBB in both Transwell-based static model and healthy mice for targeting glioma tumors [107]. H-ferritin enters and exits the BBB via an endosomal compartment and specifically targets and enters glioma cells, and consequently kills glioma tumor cells. Such kinds of transcytosis and therapeutic effects occur on using an in vitro NVU model or a neuronal disorder model. Second, in situ and non-destructive monitoring of biological changes, including BBB integrity, neural function, and inflammatory secretomes, should be functionalized on the neurovascular model for high-throughput and precise analysis against neurotoxic materials or drug candidates. The current cell-based signal-monitoring platforms are mostly based on immunofluorescence, which warrants a cellular fixation process that cannot be performed during in situ monitoring of neurons. The basis of communication in NVU is electrical signals; therefore, neuron functionality can be monitored via detection of electrical and electrochemical signals in cells as well as neurotransmitter secretions [108,109,110,111]. Based on the physiological phenomenon occurring in NVUs, electrical and electrochemical-based sensing and screening platforms can be contemplated as an integrative functional unit, which facilitates the development of new drugs and in turn providing more efficient screening platforms [112].

**Figure 6:**
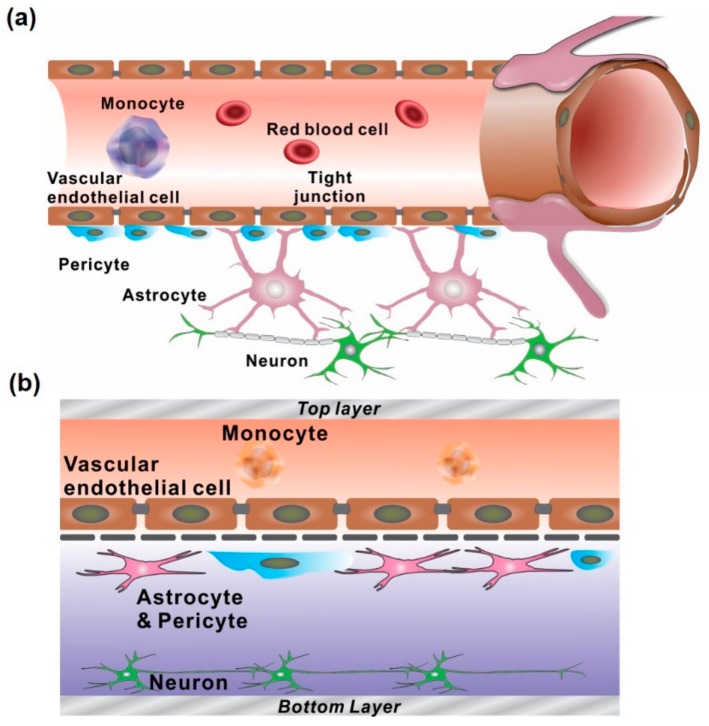
Comparison of the in vivo and in vitro neurovascular unit structure. (**a**) The schematics of the in vivo human neurovascular unit. (**b**) Representative in vitro microfluidic-integrated NVU models with vertically-aligned two microfluidic channels.

In this review, we discussed the recent developments of in vitro BBB models and NVU models that mimic the human microphysiological system as an alternative to the existing animal and in vitro cell culture models. In recent times, there are considerable shortcomings in microfluidic chip technologies pertaining to the challenges of simulating physiological features similar to those associated with an in vivo human NVU (Figure 6). However, the integration of a sensing platform with drug evaluation will offer a breakthrough platform for developing effective therapeutic agents and facilitating early diagnosis of various neuronal disorders, which can, in turn, improve biomedical, pharmaceutical, and clinical applications.

## Figures and Tables

**Table 1:** Advantages and limitations of current in vitro BBB models.

Structure		Advantage	Limitation	Function	Reference
**Static BBB Model**	2D static Transwell	High-integrity of BBB using hPSCs-derived vascular endothelial cells for BBB formation	No fluidic flow and shear stress	Monitoring BBB integrity such as TEER	[37]
	3D printed holder and electrospun PLGA Mesh for BBB	Significant barrier integrity with tight junction protein expression by PLGA nanofiber mesh	No fluidic flow and shear stress Too thick to mimic the membrane below BBB	Testing anti-brain tumor drugs (paclitaxel and bortezomib) and a neurotoxic peptide (amyloid β 1-42)	[38]
	2D static filter-free BBB model	Prevention of adhesion of numerous types of nanoparticles to the membrane filter	No fluidic flow and shear stress Not to use astrocyte to form BBB	Observation of transendothelial delivery of PEG-P(CL-g-TMC) polymersomes	[39]
	2D static Transwell	Barrier enhancement and reduced transcytosis by iPSC-derived BMECs, astrocytes, pericyte, and neurons to form an isogenic human model.	No fluidic flow and shear stress	Measurement of TEER value and permeability with iPSC-derived BMECs, astrocytes, and pericyte	[40]
**Microfluidic-integrated BBB Model**	Horizontal-aligned BBB models	Easy-to-make BBB model with astrocyte, endothelium, and neuron with 3D hydrogel structure	A low contact area between neuronal and vascular channels	Measurement of TEER, permeability, efflux activity Applicable to mechanical stress and the change of BBB integrity	[41,42,43]
	Vertical-aligned BBB models	Induction of crosstalk between neuronal cells and vascular endothelium via the porous membrane	Relatively hard-to-make the vertical structure comparing horizontal model A low contact area between neuronal and vascular channels	Monitoring TEER value Evaluation of drug permeability and cytotoxicity of CNS drug candidates Pulse generation for enhanced permeability	[44,45,46,47]
	Tubular structure	Structural similarity of the blood vessel in BBB with 3D neuronal structure Induction of biological membrane	Insufficient factors to mimic the in vitro BBB The difficulty of maintaining for an extended period	Monitoring TEER value and permeability Observation of increased leukocyte adhesion on endothelium	[48,49,50]

**Table 2:** Advantages and limitations of current in vitro microfluidic ND models

Structure		Advantage	Limitation	Function	Reference
**2D Neurological Disorder Model**	Axonal injury model	Easy to mimic, simple to perform and versatile	High precision requires a more complex microfluidic device Maybe an inappropriate model for SCIs study	Simple methods employed for disconnection and regeneration of axons Myelination along with the axonal growth via oligodendrocyte	[64,65,66]
	ALS model	Co-culture systems to form good NMJ Simplified and efficient system to demonstrate formation of NMJ	Not using 3D ECM materials	Drug screening platform for neurodegenerative disease involving NMJ	[70,79]
	AD model	AD was induced simply by applying Aβ	Not precise model for AD Short maintenance period when comparing Aβ deposition time	Analysis of neuronal cell viability towards Aβ Microglia migration assay by applying Aβ Mimicry of the interstitial flow in the brain	[67,80]
**3D Neurological Disorder Model**	AD model	3D Human Tri-Culture System Modeling AD provides critical AD features such as Aβ aggregation, p-tau accumulation, and neuroinflammatory and neurotoxic activity The precise model recapitulates most AD features	Complex process involved in the generation of the human AD tri-culture model Proper control of critical factors such as pH, oxygen tension, etc. is difficult in 3D culture	Enables the study of microglia recruitment, neuroinflammatory response and neuron/astrocyte damages	[75,76]
	ALS model	3D muscular strips and motor neuron elongation in 3D to generate functional NMJ	No information on pathogenic roles of astrocytes, glia and other factors in ALS	Optogenetic stimulation enabled contraction Can serve as ALS disease model with ALS-patient derived cells	[77]
	PD model	PD-specific dopaminergic neurons in 3d microfluidics The 3D approach reveals robust endophenotype	Only neurons were assessed Other cell types which contribute to PD pathology were ignored	In-vitro models for patient stratification and personalized drug development	[78]

**Table 3:** Advantages and limitations of in vitro neurovascular unit and disorder models.

Structure/Condition		Advantage	Limitation	Function	Reference
**Neurovascular Unit Models**	Horizontal -aligned neurovascular unit (heuron, astrocyte, endothelium)	Enable quantitative assessment of neuronal responses	Mixed cell origin make it difficult to mimic human (Rat astrocyte and neuron, human cerebral microvascular endothelium)	Monitoring TEER value and permeability	[81]
	Horizontal-aligned neural network (astrocyte, endothelium)	Providing a stable surface for culturing human cells by dopamine coating	No pericytes and neurons Human umbilical vein endothelial cells are not brain endothelium	Monitoring TEER value and permeability	[82]
	Vertical -aligned neurovascular unit (neuron, astrocyte, pericyte, endothelium)	Allowing cell-to-cell communication	Incorrect TEER value compared to Transwell system due to narrow microchannel	Monitoring TEER value and permeability	[83]
	Vertical -aligned neurovascular unit (astrocyte, pericyte, endothelium)	Robustness of in vitro model of the BBB by tri-culture model	No neurons in device	Showing functional expression of the P-gp efflux pump	[84]
	Static Transwell model (astrocyte, neuron, endothelium)	Use the human-derived stem cells for forming neurovascular unit	Not to use fluidic flow	Transport study regarding several neuronal drugs	[85,86]
	Horizontal -aligned neurovascular unit (neural stem cell, endothelium)	Providing the information of NSC-vascular niche	No pericytes, astrocyte, and neurons	Notch effectors regulate differentiation and self-renewal, more actively around endothelium	[87]
	Vertical -aligned neurovascular unit (neuron, astrocyte, pericyte, endothelium)	Offering perfusable and selective microvasculature	No neurons in device	Induction of microvascular network in 3D neural network	[88]
**Neuroinflammation Models**	Vertical -aligned neurovascular unit, stimulated with TNF-α	Enable to check the microglia activation against TNF-α	No 3D culture of neural cells	Monitoring TEER value and permeability by TNF-α treatment	[93]
	Tubular structured BBB stimulated with TNF-α and oxygen-glucose deprivation	Providing immune cell activation in ischemic and inflamed condition	Only endothelium and BBB structure	Monitoring TEER value and permeability of inflamed and recovered state	[94]
	Tubular structured neurovascular unit stimulated with TNF-α	Simple model to identify contributions of neuronal cells to the neuroinflammation	No neurons and immune cells in device	Measurement of granulocyte colony-stimulating factor and interleukin-6	[95]
**Neurological Disorder Models**	Horizontal -aligned neurovascular unit for organophosphate (OP) toxicity screening	Offering OP testing platform by emulating hyper-cholinergic activity in device	No pericyte in device	Monitoring acetylcholinesterase activity	[96]
	Horizontal -aligned neurovascular unit for brain metastasis	Enable to examine brain metastasis of cancer and their therapeutic responses	No neurons and immune cells in device	Detection of transcytosis of diverse cancer cells and evaluate drug efficacy	[97]
	Neurospheroid for mimicry of AD in microfluidics	Induction of neuronal degeneration by Aβ with interstitial flow	No BBB structure with barrier function	Evaluation of Aβ toxicity by immunostaining	[75]

## References

[1] World Health Organization (2006). Neurological Disorders: Public Health Challenges.

[2] Lashuel H.A., Hartley D., Petre B.M., Walz T., Lansbury P.T. (2002). Neurodegenerative disease: Amyloid pores from pathogenic mutations. Nature.

[3] Hardy J., Gwinn-Hardy K. (1998). Genetic classification of primary neurodegenerative disease. Science.

[4] Bertram L., Tanzi R.E. (2005). The genetic epidemiology of neurodegenerative disease. J. Clin. Investig..

[5] Galimberti D., Scarpini E. (2018). Neurodegenerative Diseases.

[6] Lang A.E., Espay A.J. (2018). Disease Modification in Parkinson’s Disease: Current Approaches, Challenges, and Future Considerations. Mov. Disord..

[7] Kieburtz K., Reilmann R., Olanow C.W. (2018). Huntington’s disease: Current and future therapeutic prospects. Mov. Disord..

[8] Cummings J., Lee G., Ritter A., Zhong K. (2018). Alzheimer’s disease drug development pipeline: 2018. Alzheimer’s Dement. Transl. Res. Clin. Interv..

[9] Mitchell B.F., Taggart M.J. (2009). Are animal models relevant to key aspects of human parturition?. Am. J. Physiol. Regul. Integr. Comp. Physiol..

[10] Esmon C.T. (2004). Why do animal models (sometimes) fail to mimic human sepsis?. Crit. Care Med..

[11] Shanks N., Greek R., Greek J. (2009). Are animal models predictive for humans?. Philos. Ethics. Hum. Med..

[12] Hawkins B.T., Davis T.P. (2005). The blood-brain barrier/neurovascular unit in health and disease. Pharm. Rev..

[13] Zlokovic B.V. (2010). Neurodegeneration and the neurovascular unit. Nat. Med..

[14] Lok J., Gupta P., Guo S., Kim W.J., Whalen M.J., van Leyen K., Lo E.H. (2007). Cell-cell signaling in the neurovascular unit. Neurochem. Res..

[15] Lecrux C., Hamel E. (2011). The neurovascular unit in brain function and disease. Acta Physiol. Oxf..

[16] Vissapragada R., Contreras M.A., da Silva C.G., Kumar V.A., Ochoa A., Vasudevan A., Selim M.H., Ferran C., Thomas A.J. (2014). Bidirectional crosstalk between periventricular endothelial cells and neural progenitor cells promotes the formation of a neurovascular unit. Brain. Res..

[17] Banks W.A., Kovac A., Morofuji Y. (2018). Neurovascular unit crosstalk: Pericytes and astrocytes modify cytokine secretion patterns of brain endothelial cells. J. Cereb. Blood Flow Metab..

[18] Miyamoto N., Pham L.D.D., Seo J.H., Kim K.W., Lo E.H., Arai K. (2014). Crosstalk between cerebral endothelium and oligodendrocyte. Cell. Mol. Life Sci..

[19] Topper J.N., Gimbrone M.A. (1999). Blood flow and vascular gene expression: Fluid shear stress as a modulator of endothelial phenotype. Mol. Med. Today.

[20] Yamamoto K., Sokabe T., Watabe T., Miyazono K., Yamashita J.K., Obi S., Ohura N., Matsushita A., Kamiya A., Ando J. (2005). Fluid shear stress induces differentiation of Flk-1-positive embryonic stem cells into vascular endothelial cells in vitro. Am. J. Physiol. Heart Circ. Physiol..

[21] Wang H., Riha G.M., Yan S., Li M., Chai H., Yang H., Yao Q., Chen C. (2005). Shear stress induces endothelial differentiation from a murine embryonic mesenchymal progenitor cell line. Arter. Thromb. Vasc. Biol..

[22] Chou C.H., Sinden J.D., Couraud P.O., Modo M. (2014). In vitro modeling of the neurovascular environment by coculturing adult human brain endothelial cells with human neural stem cells. PLoS ONE.

[23] Xue Q., Liu Y., Qi H., Ma Q., Xu L., Chen W., Chen G., Xu X. (2013). A novel brain neurovascular unit model with neurons, astrocytes and microvascular endothelial cells of rat. Int. J. Biol. Sci..

[24] ElAli A., Theriault P., Rivest S. (2014). The role of pericytes in neurovascular unit remodeling in brain disorders. Int. J. Mol. Sci..

[25] Becerra-Calixto A., Cardona-Gomez G.P. (2017). The Role of Astrocytes in Neuroprotection after Brain Stroke: Potential in Cell Therapy. Front. Mol. Neurosci..

[26] Pardridge W.M. (2005). The blood-brain barrier: Bottleneck in brain drug development. NeuroRx.

[27] Furtado D., Björnmalm M., Ayton S., Bush A.I., Kempe K., Caruso F. (2018). Overcoming the Blood–Brain Barrier: The Role of Nanomaterials in Treating Neurological Diseases. Adv. Mater..

[28] Abbott N.J., Patabendige A.A., Dolman D.E., Yusof S.R., Begley D.J. (2010). Structure and function of the blood-brain barrier. Neurobiol. Dis..

[29] Jiao H., Wang Z., Liu Y., Wang P., Xue Y. (2011). Specific role of tight junction proteins claudin-5, occludin, and ZO-1 of the blood-brain barrier in a focal cerebral ischemic insult. J. Mol. Neurosci..

[30] Shawahna R., Uchida Y., Decleves X., Ohtsuki S., Yousif S., Dauchy S., Jacob A., Chassoux F., Daumas-Duport C., Couraud P.-O. (2011). Transcriptomic and quantitative proteomic analysis of transporters and drug metabolizing enzymes in freshly isolated human brain microvessels. Mol. Pharm..

[31] Ballabh P., Braun A., Nedergaard M. (2004). The blood-brain barrier: An overview: Structure, regulation, and clinical implications. Neurobiol. Dis..

[32] Armulik A., Genove G., Mae M., Nisancioglu M.H., Wallgard E., Niaudet C., He L., Norlin J., Lindblom P., Strittmatter K. (2010). Pericytes regulate the blood-brain barrier. Nature.

[33] Yao Y., Chen Z.L., Norris E.H., Strickland S. (2014). Astrocytic laminin regulates pericyte differentiation and maintains blood brain barrier integrity. Nat. Commun..

[34] Zlokovic B.V. (2008). The blood-brain barrier in health and chronic neurodegenerative disorders. Neuron.

[35] Wang Y.I., Abaci H.E., Shuler M.L. (2017). Microfluidic blood-brain barrier model provides in vivo-like barrier properties for drug permeability screening. Biotechnol. Bioeng..

[36] Ohtsuki S., Ikeda C., Uchida Y., Sakamoto Y., Miller F., Glacial F., Decleves X., Scherrmann J.-M., Couraud P.-O., Kubo Y. (2012). Quantitative targeted absolute proteomic analysis of transporters, receptors and junction proteins for validation of human cerebral microvascular endothelial cell line hCMEC/D3 as a human blood–brain barrier model. Mol. Pharm..

[37] Weksler B., Romero I.A., Couraud P.-O. (2013). The hCMEC/D3 cell line as a model of the human blood brain barrier. Fluids Barriers CNS.

[38] D’Aversa T.G., Eugenin E.A., Lopez L., Berman J.W. (2013). Myelin basic protein induces inflammatory mediators from primary human endothelial cells and blood-brain barrier disruption: Implications for the pathogenesis of multiple sclerosis. Neuropathol. Appl. Neurobiol..

[39] Qian T., Maguire S.E., Canfield S.G., Bao X., Olson W.R., Shusta E.V., Palecek S.P. (2017). Directed differentiation of human pluripotent stem cells to blood-brain barrier endothelial cells. Sci. Adv..

[40] Qi D., Wu S., Lin H., Kuss M.A., Lei Y., Krasnoslobodtsev A., Ahmed S., Zhang C., Kim H.J., Jiang P. (2018). Establishment of a Human iPSC-and Nanofiber-Based Microphysiological Blood–Brain Barrier System. ACS Appl. Mater. Interfaces.

[41] De Jong E., Williams D.S., Abdelmohsen L., Van Hest J.C.M., Zuhorn I.S. (2018). A filter-free blood-brain barrier model to quantitatively study transendothelial delivery of nanoparticles by fluorescence spectroscopy. J. Control. Release.

[42] Stebbins M.J., Gastfriend B.D., Canfield S.G., Lee M.S., Richards D., Faubion M.G., Li W.J., Daneman R., Palecek S.P., Shusta E.V. (2019). Human pluripotent stem cell-derived brain pericyte-like cells induce blood-brain barrier properties. Sci. Adv..

[43] Prabhakarpandian B., Shen M.C., Nichols J.B., Mills I.R., Sidoryk-Wegrzynowicz M., Aschner M., Pant K. (2013). SyM-BBB: A microfluidic Blood Brain Barrier model. Lab Chip.

[44] Deosarkar S.P., Prabhakarpandian B., Wang B., Sheffield J.B., Krynska B., Kiani M.F. (2015). A Novel Dynamic Neonatal Blood-Brain Barrier on a Chip. PLoS ONE.

[45] Partyka P.P., Godsey G.A., Galie J.R., Kosciuk M.C., Acharya N.K., Nagele R.G., Galie P.A. (2017). Mechanical stress regulates transport in a compliant 3D model of the blood-brain barrier. Biomaterials.

[46] Booth R., Kim H. (2012). Characterization of a microfluidic in vitro model of the blood-brain barrier (μBBB). Lab A Chip.

[47] Booth R., Kim H. (2014). Permeability analysis of neuroactive drugs through a dynamic microfluidic in vitro blood–brain barrier model. Ann. Biomed. Eng..

[48] Shao X., Gao D., Chen Y., Jin F., Hu G., Jiang Y., Liu H. (2016). Development of a blood-brain barrier model in a membrane-based microchip for characterization of drug permeability and cytotoxicity for drug screening. Anal. Chim. Acta.

[49] Bonakdar M., Graybill P.M., Davalos R.V. (2017). A microfluidic model of the blood-brain barrier to study permeabilization by pulsed electric fields. RSC Adv..

[50] Kim J.A., Kim H.N., Im S.K., Chung S., Kang J.Y., Choi N. (2015). Collagen-based brain microvasculature model in vitro using three-dimensional printed template. Biomicrofluidics.

[51] Marino A., Tricinci O., Battaglini M., Filippeschi C., Mattoli V., Sinibaldi E., Ciofani G. (2018). A 3D Real-Scale, Biomimetic, and Biohybrid Model of the Blood-Brain Barrier Fabricated through Two-Photon Lithography. Small.

[52] Linville R.M., DeStefano J.G., Sklar M.B., Xu Z., Farrell A.M., Bogorad M.I., Chu C., Walczak P., Cheng L., Mahairaki V. (2019). Human iPSC-derived blood-brain barrier microvessels: Validation of barrier function and endothelial cell behavior. Biomaterials.

[53] Bergmann S., Lawler S.E., Qu Y., Fadzen C.M., Wolfe J.M., Regan M.S., Pentelute B.L., Agar N.Y.R., Cho C.F. (2018). Blood-brain-barrier organoids for investigating the permeability of CNS therapeutics. Nat. Protoc..

[54] Cho C.-F., Wolfe J.M., Fadzen C.M., Calligaris D., Hornburg K., Chiocca E.A., Agar N.Y., Pentelute B.L., Lawler S.E. (2017). Blood-brain-barrier spheroids as an in vitro screening platform for brain-penetrating agents. Nat. Commun..

[55] Carey J. (1990). Brain Facts: A Primer on the Brain and Nervous System.

[56] Chesselet M.-F., Carmichael S.T. (2012). Animal Models of Neurological Disorders.

[57] Jucker M. (2010). The benefits and limitations of animal models for translational research in neurodegenerative diseases. Nat. Med..

[58] Rothbauer M., Zirath H., Ertl P. (2018). Recent advances in microfluidic technologies for cell-to-cell interaction studies. Lab Chip.

[59] Walker G.M., Zeringue H.C., Beebe D.J. (2004). Microenvironment design considerations for cellular scale studies. Lab Chip.

[60] Choi J.H., Cho H.Y., Choi J.W. (2017). Microdevice Platform for In Vitro Nervous System and Its Disease Model. Bioengineering.

[61] Campenot R.B. (1977). Local control of neurite development by nerve growth factor. Proc. Natl. Acad. Sci. USA.

[62] Whitesides G.M. (2006). The origins and the future of microfluidics. Nature.

[63] Park J.W., Kim H.J., Kang M.W., Jeon N.L. (2013). Advances in microfluidics-based experimental methods for neuroscience research. Lab Chip.

[64] Osaki T., Shin Y., Sivathanu V., Campisi M., Kamm R.D. (2018). In Vitro Microfluidic Models for Neurodegenerative Disorders. Adv. Healthc. Mater..

[65] Shrirao A.B., Kung F.H., Omelchenko A., Schloss R.S., Boustany N.N., Zahn J.D., Yarmush M.L., Firestein B.L. (2018). Microfluidic platforms for the study of neuronal injury in vitro. Biotechnol. Bioeng..

[66] Hosmane S., Fournier A., Wright R., Rajbhandari L., Siddique R., Yang I.H., Ramesh K.T., Venkatesan A., Thakor N. (2011). Valve-based microfluidic compression platform: Single axon injury and regrowth. Lab Chip.

[67] Kim H.J., Park J.W., Byun J.H., Vahidi B., Rhee S.W., Jeon N.L. (2012). Integrated microfluidics platforms for investigating injury and regeneration of CNS axons. Ann. Biomed. Eng..

[68] Neukomm L.J., Freeman M.R. (2014). Diverse cellular and molecular modes of axon degeneration. Trends Cell Biol..

[69] Cho H., Hashimoto T., Wong E., Hori Y., Wood L.B., Zhao L., Haigis K.M., Hyman B.T., Irimia D. (2013). Microfluidic chemotaxis platform for differentiating the roles of soluble and bound amyloid-β on microglial accumulation. Sci. Rep..

[70] Song H.L., Shim S., Kim D.H., Won S.H., Joo S., Kim S., Jeon N.L., Yoon S.Y. (2014). β-Amyloid is transmitted via neuronal connections along axonal membranes. Ann. Neurol..

[71] Kunze A., Lengacher S., Dirren E., Aebischer P., Magistretti P.J., Renaud P. (2013). Astrocyte-neuron co-culture on microchips based on the model of SOD mutation to mimic ALS. Integr. Biol. Camb.

[72] Southam K.A., King A.E., Blizzard C.A., McCormack G.H., Dickson T.C. (2013). Microfluidic primary culture model of the lower motor neuron-neuromuscular junction circuit. J. Neurosci. Methods.

[73] Zhuang P., Sun A.X., An J., Chua C.K., Chew S.Y. (2018). 3D neural tissue models: From spheroids to bioprinting. Biomaterials.

[74] Lee C.T., Bendriem R.M., Wu W.W., Shen R.F. (2017). 3D brain Organoids derived from pluripotent stem cells: Promising experimental models for brain development and neurodegenerative disorders. J. Biomed. Sci..

[75] Haring A.P., Sontheimer H., Johnson B.N. (2017). Microphysiological Human Brain and Neural Systems-on-a-Chip: Potential Alternatives to Small Animal Models and Emerging Platforms for Drug Discovery and Personalized Medicine. Stem Cell Rev. Rep..

[76] Wang Y., Wang L., Zhu Y., Qin J. (2018). Human brain organoid-on-a-chip to model prenatal nicotine exposure. Lab Chip.

[77] Park J., Lee B.K., Jeong G.S., Hyun J.K., Lee C.J., Lee S.H. (2015). Three-dimensional brain-on-a-chip with an interstitial level of flow and its application as an in vitro model of Alzheimer’s disease. Lab Chip.

[78] Park J., Wetzel I., Marriott I., Dréau D., D’Avanzo C., Kim D.Y., Tanzi R.E., Cho H. (2018). A 3D human triculture system modeling neurodegeneration and neuroinflammation in Alzheimer’s disease. Nat. Neurosci..

[79] Bolognin S., Fossepre M., Qing X., Jarazo J., Scancar J., Moreno E.L., Nickels S.L., Wasner K., Ouzren N., Walter J. (2019). 3D Cultures of Parkinson’s Disease-Specific Dopaminergic Neurons for High Content Phenotyping and Drug Testing. Adv. Sci. Weinh.

[80] Uzel S.G., Platt R.J., Subramanian V., Pearl T.M., Rowlands C.J., Chan V., Boyer L.A., So P.T., Kamm R.D. (2016). Microfluidic device for the formation of optically excitable, three-dimensional, compartmentalized motor units. Sci. Adv..

[81] Osaki T., Uzel S.G.M., Kamm R.D. (2018). Microphysiological 3D model of amyotrophic lateral sclerosis (ALS) from human iPS-derived muscle cells and optogenetic motor neurons. Sci. Adv..

[82] Banks W.A., Erickson M.A. (2010). The blood-brain barrier and immune function and dysfunction. Neurobiol. Dis..

[83] Adriani G., Ma D., Pavesi A., Kamm R.D., Goh E.L. (2017). A 3D neurovascular microfluidic model consisting of neurons, astrocytes and cerebral endothelial cells as a blood–brain barrier. Lab Chip.

[84] Nguyen P.Q.H., Duong D.D., Kwun J.D., Lee N.Y. (2019). Hybrid elastomer–plastic microfluidic device as a convenient model for mimicking the blood–brain barrier in vitro. Biomed. Microdevices.

[85] Brown J.A., Pensabene V., Markov D.A., Allwardt V., Neely M.D., Shi M., Britt C.M., Hoilett O.S., Yang Q., Brewer B.M. (2015). Recreating blood-brain barrier physiology and structure on chip: A novel neurovascular microfluidic bioreactor. Biomicrofluidics.

[86] Wang J.D., Khafagy E.-S., Khanafer K., Takayama S., ElSayed M.E. (2016). Organization of endothelial cells, pericytes, and astrocytes into a 3D microfluidic in vitro model of the blood–brain Barrier. Mol. Pharm..

[87] Appelt-Menzel A., Cubukova A., Gunther K., Edenhofer F., Piontek J., Krause G., Stuber T., Walles H., Neuhaus W., Metzger M. (2017). Establishment of a Human Blood-Brain Barrier Co-culture Model Mimicking the Neurovascular Unit Using Induced Pluri- and Multipotent Stem Cells. Stem Cell Rep..

[88] Canfield S.G., Stebbins M.J., Morales B.S., Asai S.W., Vatine G.D., Svendsen C.N., Palecek S.P., Shusta E.V. (2017). An isogenic blood-brain barrier model comprising brain endothelial cells, astrocytes, and neurons derived from human induced pluripotent stem cells. J. Neurochem..

[89] Shin Y., Yang K., Han S., Park H.J., Seok Heo Y., Cho S.W., Chung S. (2014). Reconstituting vascular microenvironment of neural stem cell niche in three-dimensional extracellular matrix. Adv. Healthc. Mater..

[90] Campisi M., Shin Y., Osaki T., Hajal C., Chiono V., Kamm R.D. (2018). 3D self-organized microvascular model of the human blood-brain barrier with endothelial cells, pericytes and astrocytes. Biomaterials.

[91] Hamer M., Chida Y. (2009). Physical activity and risk of neurodegenerative disease: A systematic review of prospective evidence. Psychol. Med..

[92] Chen W.W., Zhang X., Huang W.J. (2016). Role of neuroinflammation in neurodegenerative diseases (Review). Mol. Med. Rep..

[93] Ransohoff R.M. (2016). How neuroinflammation contributes to neurodegeneration. Science.

[94] Kempuraj D., Thangavel R., Natteru P.A., Selvakumar G.P., Saeed D., Zahoor H., Zaheer S., Iyer S.S., Zaheer A. (2016). Neuroinflammation Induces Neurodegeneration. J. Neurol. Neurosurg. Spine.

[95] Achyuta A.K., Conway A.J., Crouse R.B., Bannister E.C., Lee R.N., Katnik C.P., Behensky A.A., Cuevas J., Sundaram S.S. (2013). A modular approach to create a neurovascular unit-on-a-chip. Lab Chip.

[96] Cho H., Seo J.H., Wong K.H., Terasaki Y., Park J., Bong K., Arai K., Lo E.H., Irimia D. (2015). Three-Dimensional Blood-Brain Barrier Model for in vitro Studies of Neurovascular Pathology. Sci. Rep..

[97] Herland A., van der Meer A.D., FitzGerald E.A., Park T.E., Sleeboom J.J., Ingber D.E. (2016). Distinct Contributions of Astrocytes and Pericytes to Neuroinflammation Identified in a 3D Human Blood-Brain Barrier on a Chip. PLoS ONE.

[98] Koo Y., Hawkins B.T., Yun Y. (2018). Three-dimensional (3D) tetra-culture brain on chip platform for organophosphate toxicity screening. Sci. Rep..

[99] Xu H., Li Z., Yu Y., Sizdahkhani S., Ho W.S., Yin F., Wang L., Zhu G., Zhang M., Jiang L. (2016). A dynamic in vivo-like organotypic blood-brain barrier model to probe metastatic brain tumors. Sci. Rep..

[100] Saraiva C., Praca C., Ferreira R., Santos T., Ferreira L., Bernardino L. (2016). Nanoparticle-mediated brain drug delivery: Overcoming blood-brain barrier to treat neurodegenerative diseases. J. Control. Release.

[101] Thomford N.E., Senthebane D.A., Rowe A., Munro D., Seele P., Maroyi A., Dzobo K. (2018). Natural Products for Drug Discovery in the 21st Century: Innovations for Novel Drug Discovery. Int. J. Mol. Sci..

[102] Kang Y.J., Cutler E.G., Cho H. (2018). Therapeutic nanoplatforms and delivery strategies for neurological disorders. Nano Converg..

[103] Jeong W.J., Bu J., Kubiatowicz L.J., Chen S.S., Kim Y., Hong S. (2018). Peptide-nanoparticle conjugates: A next generation of diagnostic and therapeutic platforms?. Nano Converg..

[104] Navya P.N., Kaphle A., Srinivas S.P., Bhargava S.K., Rotello V.M., Daima H.K. (2019). Current trends and challenges in cancer management and therapy using designer nanomaterials. Nano Converg..

[105] Zhou Y., Peng Z., Seven E.S., Leblanc R.M. (2018). Crossing the blood-brain barrier with nanoparticles. J. Control. Release.

[106] Chai Z., Hu X., Wei X., Zhan C., Lu L., Jiang K., Su B., Ruan H., Ran D., Fang R.H. (2017). A facile approach to functionalizing cell membrane-coated nanoparticles with neurotoxin-derived peptide for brain-targeted drug delivery. J. Control. Release.

[107] Fan K., Jia X., Zhou M., Wang K., Conde J., He J., Tian J., Yan X. (2018). Ferritin Nanocarrier Traverses the Blood Brain Barrier and Kills Glioma. ACS Nano.

[108] Nguyen Q.T., Schroeder L.F., Mank M., Muller A., Taylor P., Griesbeck O., Kleinfeld D. (2010). An in vivo biosensor for neurotransmitter release and in situ receptor activity. Nat. Neurosci..

[109] Kim T.H., Yea C.H., Chueng S.T., Yin P.T., Conley B., Dardir K., Pak Y., Jung G.Y., Choi J.W., Lee K.B. (2015). Large-Scale Nanoelectrode Arrays to Monitor the Dopaminergic Differentiation of Human Neural Stem Cells. Adv. Mater..

[110] Shin J.W., Kim K.J., Yoon J., Jo J., El-Said W.A., Choi J.W. (2017). Silver Nanoparticle Modified Electrode Covered by Graphene Oxide for the Enhanced Electrochemical Detection of Dopamine. Sens. Basel.

[111] Park D.J., Choi J.H., Lee W.J., Um S.H., Oh B.K. (2017). Selective Electrochemical Detection of Dopamine Using Reduced Graphene Oxide Sheets-Gold Nanoparticles Modified Electrode. J. Nanosci. Nanotechnol..

[112] El-Said W.A., Yoon J., Choi J.W. (2018). Nanostructured surfaces for analysis of anticancer drug and cell diagnosis based on electrochemical and SERS tools. Nano Converg..

